# Current Status and Advances in Semen Preservation

**DOI:** 10.3390/ani13010123

**Published:** 2022-12-28

**Authors:** Anna Dziekońska, Agnieszka Partyka

**Affiliations:** 1 Department of Animal Biochemistry and Biotechnology, University of Warmia and Mazury in Olsztyn, Oczapowskiego 5, 10-719 Olsztyn, Poland; 2 Department of Reproduction and Clinic of Farm Animals, Wroclaw University of Environmental and Life Sciences, Pl. Grunwaldzki 49, 50-366 Wroclaw, Poland

## 1. Introduction

Recent advances in assisted reproductive technology (ART) have increased the effectiveness of fertility treatments. Some examples of ART include the storage of gametes and embryos, in vitro fertilization (IVF), artificial insemination (AI), and embryo transfer (ET). The storage of gametes, especially sperm, is a popular method facilitating the reproductive success of farm, companion, working, and wild-living animals. The choice of the preferred sperm-preservation method (liquid or cryopreservation) is determined by the species. Therefore, species-specific characteristics and the influence of various factors, such as storage temperature and time, or the type of diluent, have to be considered during the improvement of sperm-preservation methods. Cryopreservation of ejaculated and epididymal sperm supports the long-term preservation of valuable genetic material for reproductive purposes. The quality of preserved sperm is assessed with the use of various laboratory techniques (computer-assisted, fluorescence, or proteomic analysis). The optimal preservation technique should maintain the fertilizing ability of spermatozoa, which is evaluated based on the results of laboratory analyses and confirmed by the success rate of in vitro and AI procedures.

## 2. Current Status and Advances in Semen Preservation in This Special Issue 

Assisted reproduction technologies are applied mainly in mammals and less commonly used in birds and other taxonomic groups. Semen quality differs between species and individuals of the same species. The sperm of various animal species differ in morphology, biological parameters, and susceptibility to cryopreservation [1]. Interspecific differences in the biochemical composition of plasma membranes determine sperm freezability. In some animal species, spermatozoa are particularly sensitive to cold shock. For example, boar spermatozoa are usually stored in a liquid state [2,3], whereas ruminant sperm are generally cryopreserved [1,4]. However, liquid preservation is often the method of choice in many animal species due to its simplicity, low cost, and ability to preserve sperm fertilizing capacity. 

Numerous factors influence the quality and fertilizing ability of liquid-preserved and cryopreserved sperm. The quality of fresh semen is one such factor. Morphological defects can undermine sperm cells’ suitability for liquid preservation and cryopreservation. Henning et al. [2] reported that a high percentage of spermatozoa with morphological defects, such as cytoplasmic droplets in ejaculated boar semen (above 15%), can compromise sperm capacitation and decrease semen fertilizing capacity. 

Prolonged sperm storage in a liquid state can induce considerable changes in the morphology and function of spermatozoa. Numerous morphometric changes in the head and tail were observed in boar spermatozoa preserved in a liquid state at a temperature of 17 °C [3]. These changes were confirmed by various staining techniques (eosin-nigrosin, eosin-gentian, and SpermBlue). The applied technique also significantly influenced the severity of the observed changes. 

Semen’s suitability for cryopreservation is also determined by the animal’s age. Ejaculate volume and quality change with age and can affect sperm cells’ resistance to freezing. The quality of cryopreserved bull sperm was influenced by differences in the proteomic composition of semen [4]. The semen of adult bulls (aged 4 years) was characterized by a predominance of proteins that are associated with metabolic processes generating energy, protecting sperm against oxidative stress, and enhancing fertilizing ability, whereas the semen of juvenile bulls (up to 2 years) contained mostly cytoskeletal proteins. Differences in the proteomic composition of spermatozoa are also observed between individuals, and they can affect sperm quality. Zmudzinska et al. [5] reported differences in protein composition between the morphological structures of spermatozoa in dogs. They found that both intracellular and membrane-associated proteins of canine epididymal spermatozoa were involved in the key metabolic pathways regulating sperm functions and fertility of dogs. Selected proteins can also be used as markers of semen quality [1,5].

The choice of a suitable extender and the optimal temperature during liquid storage can enhance sperm viability and functionality. During storage, the temperature is decreased to 4–5 °C to increase the viability of sperm cells from various animal species [6,7]. Low temperatures slow down sperm metabolism and inhibit the production of toxic metabolites. However, chill-stored sperm cells are susceptible to cold shock which can damage their cell structures [1,7]. To reduce this risk, sperm extenders are enhanced with various substances, including egg yolk and plant proteins (mainly soybean), which minimize the harmful effects of cold shock. Plant proteins are increasingly recommended to decrease the content of animal-based substances in sperm extenders and to eliminate the risk of microbial contamination [7]. Substances with protective and antioxidant effects are also added to extenders to minimize the negative impact of storage on semen quality [6,7,8,9]. Alkali et al. [6] found that soybean- and egg yolk-based extenders effectively preserved the quality parameters of turkey semen, both at ambient and chilling temperatures, and reduced sperm morphological defects during storage when the extenders were supplemented with L-ascorbic acid. Extenders supplemented with nanoparticles also improved the quality of turkey spermatozoa preserved in a liquid state. Orzołek et al. [8] analyzed the quality of turkey spermatozoa stored for up to 48 h in extenders containing zinc nanoparticles (ZnNPs) and manganese nanoparticles (MnNPs). The addition of ZnNPs decreased mitochondrial membrane potential and mitochondrial activity in turkey sperm. However, the supplementation of turkey ejaculates with a lower concentration of MnNPs (25 μM) enhanced membrane integrity, membrane mitochondrial potential, and superoxide dismutase activity, especially during prolonged storage [8].

Cervid spermatozoa are preserved with the use of extenders designed for other ruminants (cattle and sheep). Differences in extender composition can significantly affect the viability and functionality of European red deer epididymal spermatozoa stored at a temperature of 5 °C [7]. An egg yolk-based extender was most effective in preserving the motility and functionality of red deer spermatozoa up to the ninth day of storage at a temperature of 5 °C. The viability and quality of sperm stored in extenders designed for storing sperm in both liquid and cryopreserved states were significantly lower, which can be attributed to the presence of glycerol.

Cryopreservation is the most effective technique for the long-term conservation of valuable animal genetic resources [1,9,10]. However, cryopreservation negatively affects sperm structure and function, and new technological solutions are being sought to minimize cryogenic-induced damage. Cryoprotectants such as glycerol, egg yolk, or soy lecithin (SL) are added to semen extenders to improve the quality of post-thaw sperm in various livestock species [1]. The quality and fertilizing ability of frozen/thawed rooster sperm were improved by supplementing the freezing extender with a low concentration of 3–6% dimethylacetamide (DMA) [11]. Moreover, anti-freeze-associated genes such as heat shock protein 70 (HSP70) and ras homolog family member A (RHOA) are down-regulated at high concentrations of DMA. The semen of Czech Golden Spotted Hen roosters was also effectively preserved in extenders supplemented with 9% N-methylacetamide (NMA) [12].

In addition, cryopreservation contributes to oxidative stress which leads to the production of reactive oxygen species (ROS) that can damage various structures in sperm cells [1]. The addition of antioxidants to freezing extenders can minimize oxidative stress. Extender supplementation with 2 mM proline enhanced the quality of post-thaw goat sperm (plasma membrane integrity, motility, antioxidant status) by reducing the severity of oxidative stress during cryopreservation [13]. The addition of 1% nano-SL to the freezing extender improved the quality and fertilizing capacity of rooster sperm after AI [9].

Cryopreservation is most often applied in mammals and birds used as livestock [1]. This preservation technique has been optimized for farm animals, and further research is needed to develop the most appropriate cryopreservation methods for non-domesticated and wild-living animals.

Castillo et al. [14] proposed an original method for collecting semen from wild birds (pheasants) without restricting their free movement. They evaluated the qualitative parameters of sperm from males fed an antioxidant-enriched diet (supplemented with α-tocopheryl-acetate and selenium) and analyzed the fertilizing capacity of cryopreserved semen in vivo.

Little is known about the effectiveness of sperm cryopreservation in other taxonomic groups, such as reptiles. Sperm conservation was moderately successful in several snake species. However, research has shown that selected cryoprotective agents and post-thaw treatments can enhance the quality of cryopreserved sperm from the endangered Louisiana pine snake (*Pituophis ruthveni*) [10]. The results of the cited study can contribute to the improvement of cryopreservation methods for endangered snake species.

## 3. Conclusions

Numerous factors, including species-specific differences, quality of fresh semen, conservation methods (including extender composition, storage temperature and time), and the animal’s age, have to be considered in the process of developing effective semen preservation techniques. Each of these factors affects the quality of preserved sperm. The articles published in this Special Issue have significantly expanded current knowledge on liquid preservation and cryopreservation of sperm from various animal species (representing three taxonomic groups: mammals, birds, and reptiles). The presented studies have also contributed new information about the quality and preservation of sperm from wild-living animals.

## Data Availability

Not applicable.

## References

[1] Akhtar M., Ma Q., Li Y., Chai W., Zhang Z., Li L., Wang C. (2022). Effect of Sperm Cryopreservation in Farm Animals Using Nanotechnology. Animals.

[2] Henning H., Luther A., Waberski D. (2021). A High Incidence of Sperm with Cytoplasmic Droplets Affects the Response to Bicarbonate in Preserved Boar Semen. Animals.

[3] Szablicka D., Wysokińska A., Pawlak A., Roman K. (2022). Morphometry of Boar Spermatozoa in Semen Stored at 17 °C-The Influence of the Staining Technique. Animals.

[4] Westfalewicz B., Słowińska M., Judycka S., Ciereszko A., Dietrich M. (2021). Comparative Proteomic Analysis of Young and Adult Bull (Bos taurus) Cryopreserved Semen. Animals.

[5] Zmudzinska A., Bromke M., Strzezek R., Zielinska M., Olejnik B., Mogielnicka-Brzozowska M. (2022). Proteomic Analysis of Intracellular and Membrane-Associated Fractions of Canine (Canis lupus familiaris) Epididymal Spermatozoa and Sperm Structure Separation. Animals.

[6] Alkali I., Asuku S., Colombo M., Bukar M., Waziri M., Luvoni G. (2022). Spermatozoa Survival in Egg Yolk-Based and Soybean-Based Extenders at Ambient and Chilling Temperature in Domestic Turkeys (Meleagris gallopavo). Animals.

[7] Dziekońska A., Neuman N., Burdal K., Wiszniewska-Łaszczych A., Bogdaszewski M. (2022). The Effect of Different Extenders on the Quality Characteristics of European Red Deer Epididymal Sperm Stored at 5 C. Animals.

[8] Orzołek A., Rafalska K., Otowska W., Kordan W., Korzekwa A., Kozłowski K. (2021). Influence of Zinc and Manganese Nanoparticles on Selected Parameters of Turkey Spermatozoa Stored in a Liquid State at 4 °C. Animals.

[9] Sun L., He M., Wu C., Zhang S., Dai J., Zhang D. (2021). Beneficial Influence of Soybean Lecithin Nanoparticles on Rooster Frozen–Thawed Semen Quality and Fertility. Animals.

[10] Sandfoss M., Cantrell J., Roberts B., Reichling S. (2022). Cryopreservation of Sperm from an Endangered Snake with Tests of Post-Thaw Incubation in Caffeine. Animals.

[11] Mehaisen G., Elomda A., Hamad S., Ghaly M., Sun Y., Li Y., Zong Y., Chen J., Partyka A., Nazmi A. (2022). Effect of Dimethylacetamide Concentration on Motility, Quality, Antioxidant Biomarkers, Anti-Freeze Gene Expression, and Fertilizing Ability of Frozen/Thawed Rooster Sperm. Animals.

[12] Petričáková K., Janošíková M., Ptáček M., Zita L., Savvulidi F., Partyka A. (2022). Comparison of Commercial Poultry Semen Extenders Modified for Cryopreservation Procedure in the Genetic Resource Program of Czech Golden Spotted Hen. Animals.

[13] Zhang W., Min L., Li Y., Lang Y., Hoque S., Adetunji A., Zhu Z. (2022). Beneficial Effect of Proline Supplementation on Goat Spermatozoa Quality during Cryopreservation. Animals.

[14] Castillo A., Lenzi C., Pirone A., Baglini A., Russo C., Soglia D., Schiavone A., Marzoni Fecia di Cossato M. (2021). From the Semen Collection Method to the Hatchlings: The Use of Cryopreserved Sperm from Pheasants Fed an Antioxidant-Enriched Diet. Animals.

